# Thyroid storm complicated by corpus callosum infarction in a young patient: A case report and literature review

**DOI:** 10.1097/MD.0000000000030318

**Published:** 2022-08-26

**Authors:** Yunjia Cai, Linan Ren, Xinming Liu, Chen Li, Xiaokun Gang, Guixia Wang

**Affiliations:** a Department of Endocrinology and Metabolism, The First Hospital of Jilin University, Changchun 130021, China

**Keywords:** cerebral infarction, corpus callosum, hyperthyroidism, thyroid storm (TS)

## Abstract

Thyroid storm (TS) is a rare life-threatening hypermetabolic thyrotoxicosis with an incidence of 0.57–0.76/100,000. The coexistence of TS and acute cerebral infarction is rare. Previous studies have shown that hyperthyroidism complicated by cerebral infarction mainly occurs in the intracranial basal ganglia; however, there are no reports of corpus callosum infarction. We report a case of TS complicated by cerebral infarction of the corpus callosum at our hospital.

**Patient concerns:**

A 31-year-old male patient with a history of hyperthyroidism was admitted to the hospital because of fatigue, palpitations, fever, and profuse sweating accompanied by a mild decrease in the muscle strength of the left limb. Diagnosis of a TS was confirmed by the laboratory test results. The patient’s clinical symptoms gradually improved after treatment. However, his left limb muscle strength progressively decreased, and the bilateral pathological signs were positive at the same time. Magnetic resonance imaging (MRI) of the head revealed acute cerebral infarction of the corpus callosum and pons.

**Diagnosis:**

The diagnosis was thyroid strom with acute cerebral infarction of the corpus callosum and pons and severe stenosis or occlusion of the basilar artery.

**Interventions:**

The patient was given 300 mg hydrocortisone intravenously per day, propylthiouracil tablets of 200 mg 3 times a day by nasal feeding, and 20 mg propranolol three times a day by nasal feeding. Aspirin and clopidogrel were administered to prevent platelet aggregation, and atorvastatin calcium was administered to lower lipid levels to stabilize plaques.

**Outcomes:**

The patient’s left limb muscle strength recovered to grade 4+, and he could walk beside the bed with support. Simultaneously, thyroid function was better than before.

**Lessons:**

Careful physical examination should be performed in patients with thyroid storm, and head imaging examination should be improved for the early detection of cerebral infarction.

## 1. Introduction

A thyroid storm is a rare, life-threatening endocrine emergency caused primarily by large amounts of thyroid hormones in the bloodstream that leads to multiple organ dysfunction.^[1]^ It occurs mainly in patients with untreated or inadequately treated hyperthyroidism for a long time.^[2]^ Women are more likely to develop TS, which can occur at any age.^[3]^ Cerebral infarction is a common disease that occurs primarily in middle-aged and older individuals. Cerebral infarction in young people is rare and is often secondary to moyamoya disease, cerebral arteritis, and hyperthyroidism. A recent epidemiological study found that 1% of young patients with hyperthyroidism had an ischemic stroke.^[4]^ However, thyroid storm combined with acute ischemic stroke is a rare manifestation that increases the risk of death. Early identification and timely management are key to reducing mortality and morbidity rates. The corpus callosum is located at the base of the interhemispheric fissure and is the most significant associative fiber connecting homologous site in both hemispheres.^[5]^ The corpus callosum is rich in blood supply and receives blood supply from the anterior and posterior circulation. Thus, corpus callosum infarction is rare in clinical practice. To our knowledge, there have been no reports of thyroid storm complicated by acute corpus callosum infarction worldwide and this is the first report of such a case.

## 2. Case presentation

A 31-year-old man was admitted to our hospital with symptoms of palpitations and irritability for 2 years, aggravated by fatigue, fever, and profuse sweating for 1 day. The patient presented with neck enlargement, palpitations, irritability, and proptosis 2 years ago and was diagnosed with hyperthyroidism. However, the patient did not regularly take medication or review medication. He had cold symptoms, such as nasal congestion and runny nose two days ago, but he did not investigate this further. One day prior, he developed fatigue, palpitations, fever (38.5°C), profuse sweating, and vomiting, accompanied by headache and dizziness. He denied a history of hypertension, diabetes, or coronary heart disease. He had a smoking history of 16 years (approximately 20 cigarettes per day) and opportunistic drinking. Physical examination revealed the following: body temperature, 38.5°C; pulse 143, beats/min; respiration, 22 beats/min; blood pressure, 142/92 mmHg (1 mmHg=0.133 kPa). He had mild exophthalmos, restlessness, slurred speech, dry skin, and limb tremors. Diffuse bilateral thyroid enlargement (grade II), firm palpation, poor mobility, no palpable tremor, and a vascular murmur could be heard on auscultation. He had coarse breath sounds in both lungs, moist rales at the base of both lungs, and phlegm. The cardiac boundary was normal, the heart rate was 143 beats/min, the rhythm and heart sounds were normal, and no murmurs or extra heart sounds were heard in the auscultation area of each valve. The muscle strength of the left limb was grade 4, that of the right limb was grade 5, and the pathological signs on both sides were negative. No obvious positive signs were observed during the remainder of the examination. Table 1 shows the results of the laboratory tests performed when the patient was hospitalized. Routine blood, coagulation, urine, stool, biochemistry, myocardial injury markers, high-sensitivity C-reactive protein, procalcitonin, and tumor markers were not abnormal.

**Table 1:** Laboratory date of the proband.

Variable	Test value	Reference range
Thyroid function		
TSH (uIU/ml)	**<0.0025**	0.35–4.94
FT3 (pmol/L)	**>30.72**	2.43–6.01
FT4 (pmol/L)	**>64.35**	9.01–19.05
Tg-Ab (IU/mL)	**7.35**	0–4.11
TPO-Ab (IU/mL)	**156.49**	0–5.61
TR-Ab (IU/L)	**18.070**	0–1.75
blood gas		
pH	7.44	7.35–7.45
pCO_2_ (mmHg)	37	35–48
pO_2_ (mmHg)	**70**	83–108
HCO_3_^−^ (mmol/L)	**25.1**	18.0–23.0
BE (mmol/L)	1.1	−2.0–3.0
SpO_2_ (%)	**94**	95–98
BNP(pg/ml)	**519.0**	0–125
ESR(mm/1h)	**59**	0–15
HbA1c (%)	5.7	4.0–6.0
Homocysteine (umol/L)	**15.31**	0–15
Urinary iodine (ug/L)	**489.30**	100–300

BE = base excess, BNP = pro-B-type natriuretic peptide, ESR = erythrocyte sedimentation rate, FT3 = free triiodothyronine, FT4 = free thyrocine, HbA1c = glycated haemoglobin, HCO_3_^−^ = hydrogen carbonate, pCO_2_ = partial pressure of carbon dioxide, pO_2_ = partial pressure of oxygen, SpO_2_ = Oxygen saturation, Tg-Ab = Thyroglobulin antibody, TPO-Ab = thyroid peroxidase antibodies, TR-Ab = thyrotropin receptor antibodies, TSH = thyroid stimulating hormone.

**Figure 1.**
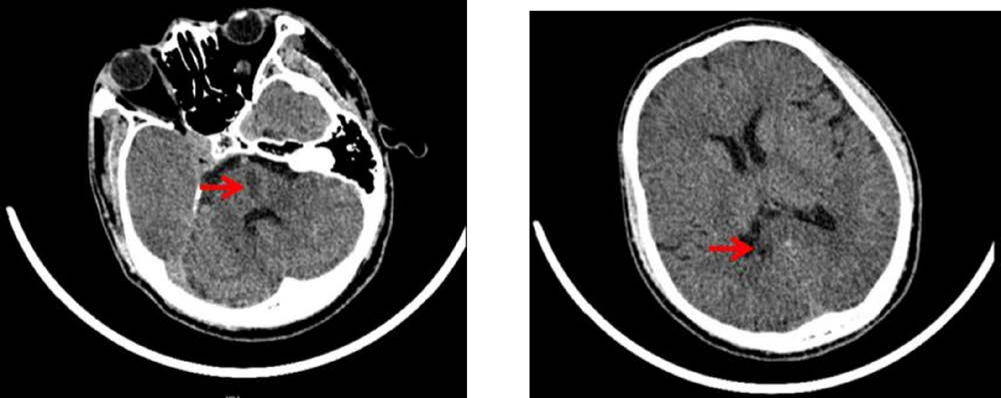
2021-11-03 Computed tomography of the head revealed suspicious hypodense shadows in the pons and corpus callosum.

**Figure 2.**
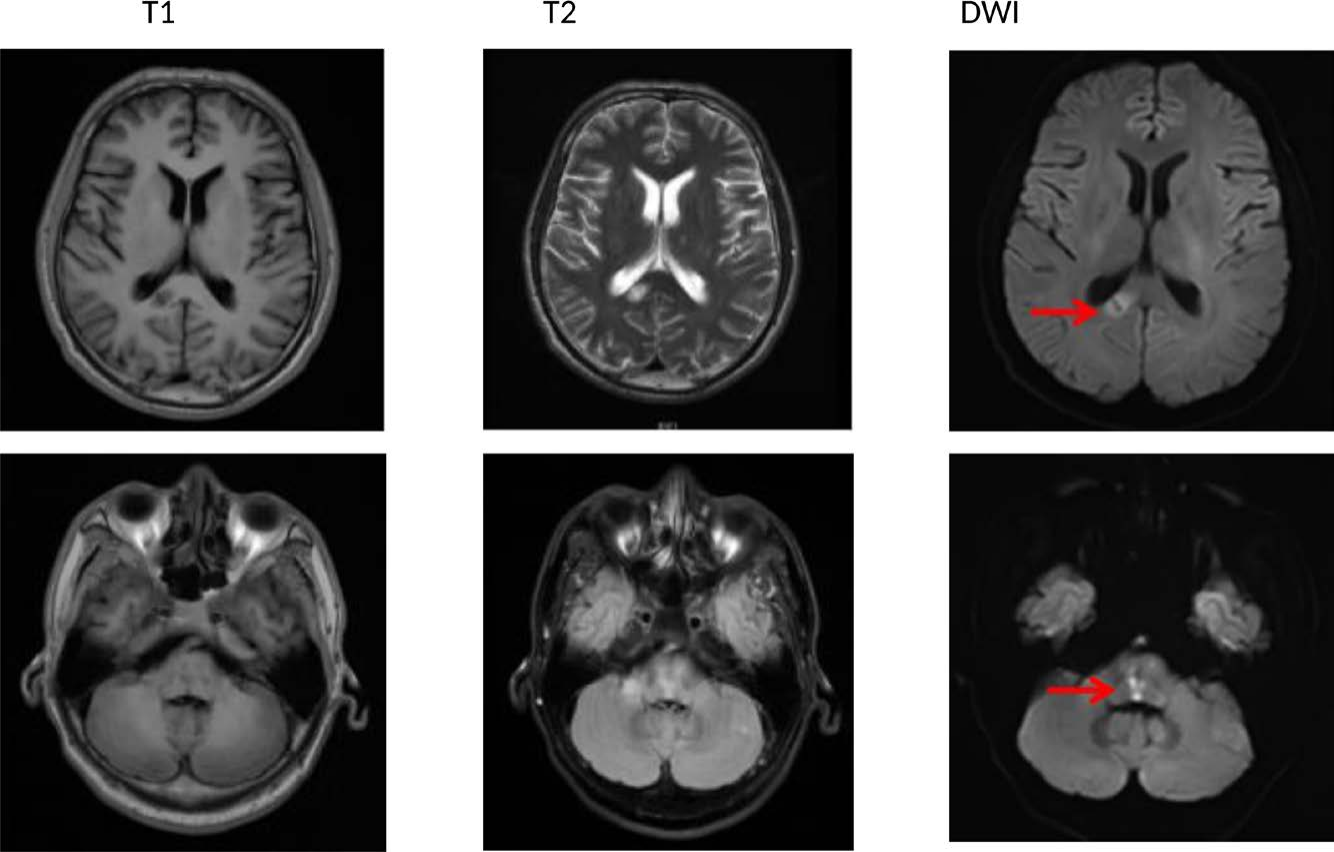
2021-11-11 The head MRI scan and diffusion examination revealed an acute and subacute lacunar infarction of the corpus callosum and pons and multiple lacunar cerebral infarctions in the brain.

**Figure 3.**
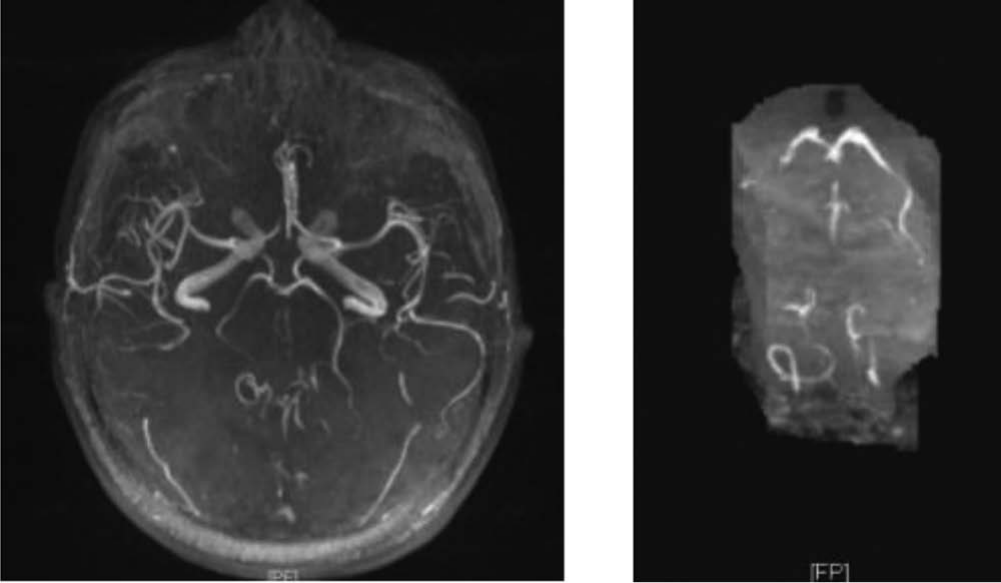
2021-11-15 The basilar artery and bilateral vertebral arteries were partially visualized but not shown locally using magnetic resonance angiography (MRA). The proximal end of the right anterior cerebral artery A2 segment was slightly visualized in the bilateral embryonic posterior cerebral arteries.

Computed tomography of the head revealed suspicious hypodense shadows in the pons and corpus callosum (Fig. 1). Computed tomography of the lungs showed sputum retention in the trachea, right main and right upper lobe bronchi, bronchitis, right upper lobe, and bilateral lower lobe inflammation. Combined with the patient’s medical history, physical examination, and related examinations, the Burch–Wartofsky scoring scale, Acute Physiology and Chronic Health Evaluation II, and sequential organ failure assessment scores were 80, 11, and 5 points, respectively. The diagnosis was thyroid storm. The patient was given 300 mg hydrocortisone intravenously per day, propylthiouracil tablets of 200 mg three times a day by nasal feeding, and 20 mg propranolol three times a day by nasal feeding. Paracetamol cooling, anti-infection, enteral nutrition, rehydration, and other symptomatic treatments were also administered. After 6 days of treatment, the patient’s general state improved, with no tremor in the limbs, first-degree thyroid enlargement, and normal body temperature and heart rate. However, neurological examination revealed that the left limb muscle strength was grade 3, the right limb muscle strength was grade 5, bilateral pathological signs were positive, bilateral nasolabial folds were symmetrical, the corners of the mouth were not skewed, the tongue was centered, and the neck was soft. His extremity muscle tone and deep and superficial sensations were both normal and symmetrical. His National Institutes of Health Stroke Scale score was 4. The head MRI scan and diffusion examination revealed an acute and subacute lacunar infarction of the corpus callosum and pons and multiple lacunar cerebral infarctions in the brain (Fig. 2). Both the vertebral arteries and intervertebral space segments showed low flow velocity and high resistance blood flow signal changes, and intracranial segment or basilar artery occlusion or severe stenosis was considered as indicated by the carotid ultrasound. The basilar artery and bilateral vertebral arteries were partially visualized but not shown locally using magnetic resonance angiography (MRA). The proximal end of the right anterior cerebral artery A2 segment was slightly visualized in the bilateral embryonic posterior cerebral arteries (Fig. 3). The diagnosis was acute cerebral infarction of the corpus callosum and pons and severe stenosis or occlusion of the basilar artery. Aspirin and clopidogrel were administered to prevent platelet aggregation, and atorvastatin calcium was administered to lower lipid levels to stabilize plaques. Simultaneously, it is necessary to improve the circulation and nutritional nerve treatment and carry out rehabilitation therapy, such as limb exercise and pulsed electric therapy. After 1 week of treatment, the patient’s left limb muscle strength recovered to grade 4+, bilateral pathological signs were negative, and he could walk beside the bed with support. Simultaneously, review thyroid function was assessed as follows: TSH, < 0.0025 uIU/mL; FT3, 8.96 pmol/L; FT4, 41.93 pmol/L. This was better than before. We recommend that the patient be taken orally 300 mg of propylthiouracil tablets daily after discharge. At the same time, aspirin and clopidogrel were administered to prevent platelet aggregation. Half a month after discharge, clinical symptoms were completely relieved at follow-up. Thyroid function was as follows: TSH, < 0.0025 uIU/mL; FT3, 20.16 pmol/L; FT4, 30.25 pmol/L. The patient was advised to continue taking propylthiouracil tablets and clopidogrel orally and to regularly review thyroid function.

## 3. Discussion

Thyroid storm is a rare endocrine emergency in clinical practice, and unrecognized and untreated thyroid storms can be fatal.^[6]^ Common triggers of TS include infection, surgery, emotional stress, iodine load, medication non-compliance, and other acute medical illnesses.^[7–11]^ According to a Japanese survey, the annual incidence of thyroid storm in hospitalized patients is 0.2/100,000, and the mortality is 10%–30%, with a 12-fold increase in mortality compared to individuals with thyrotoxicosis.^[2,3,12,13]^ Hyperthyroidism is associated with a variety of neurological diseases, such as chronic hyperthyroid myopathy, hyperthyroid hypokalemic periodic paralysis, and hyperthyroid myasthenia gravis. However, it is relatively rare to combine ischemic cerebrovascular diseases, such as cerebral infarction. A thyroid storm complicated by acute cerebral infarction is rare. The cerebral infarction site in this patient was the corpus callosum, which, to the best of our knowledge, is the first such case reported at home and abroad. In 1991, Kushima *et al.* reported the first case of hyperthyroidism complicated by acute cerebral infarction.^[14]^ In 2012, Harada *et al*. reported a case of thyroid storm complicated by acute cerebral infarction.^[15]^ We summarize the published cases of thyroid storm with acute cerebral infarction in Table 2.

**Table 2:** Summary of cases of thyroid storm complicated by acute cerebral infarction.

	Patient 1	Patient 2	Patient 3	Patient 4	Patient 5
Sex	F	F	M	F	M
Age (y)	62	49	63	43	31
Course of hyperthyroidism (mo)	0	1	0	NA	24
MRI findings	Right cerebellum	Precentral gyrus, postcentral gyrus, left frontal and posterior lobes, right parietal lobes	Posterior segment of right middle cerebral artery	Bilateral frontal and right temporal lobes	Corpus callosum and pons
Nervous system symptoms	Headache, dizziness, nausea	Quadriplegia	Left-sided hemiplegia and dysarthria	Quadriplegia and dysarthria	Left-sided hemiplegia and dysarthria
Atrial fibrillation	Y	N	Y	N	N
Thyroid function at the time of stroke (reference ranges are in parentheses)	FT3 2.90 pg/mL (1.71–3.71)	FT3 8.10 pg/dL (2–4.48)	FT3 348 ng/dL (76–181)	FT3 > 8.0 ng/mL (0.77–1.81)	FT3 > 30.72 pmol/L(2.43–6.01)
	FT4 2.92 ng/dL (0.70–1.48)	FT4 4.41 ng/dL (0.84–1.70)	FT4 5.5 ng/dL (0.6–1.6)	FT4 9.47 pmol/L (11.4–22.6)	FT4 > 64.35 pmol/L (9.01–19.05)
	TSH < 0.01 uIU/ml	TSH < 0.005 mIU/L (0.5–5)	TSH < 0.01 uIU/ml (0.35–5.5)	TSH < 0.01 uIU/ml (0.55–4.78)	TSH < 0.0025 uIU/ml (0.35–4.94)
Anti-thyroid medications	Thiamazole	Thiamazole	Propylthiouracil	Thiamazole	Propylthiouracil
Medication adherence	Good	Good	Good	Good	Good
Prognosis	Recovery	Recovery	Recovery	Recovery	Recovery
BWPS score	110	85	NA	85	80
Reference	^[15]^	^[16]^	^[17]^	^[18]^	Present case

BWPS = Burch–Wartofsky scoring scale, F = female, M = male, MRI = magnetic resonance imaging, N = no, NA = not applicable, Y = yes.

The pathogenesis of thyroid storm combined with ischemic stroke has not yet been elucidated. The literature review revealed the following: (1) thyroid hormone secretion increases significantly during a thyroid storm, sympathetic nerves are overexcited, and the concentration of catecholamines in the blood increases, causing spasms of small arteries and increased blood viscosity. Czarkowsk *et al.* found that long-term stimulation with large amounts of thyroid hormone can also reduce the toughness of blood vessels, leading to the hardening of the arteries.^[19]^ Simultaneously, thyroid hormones can promote oxidative phosphorylation, increase heat production and oxygen consumption, and cause profuse sweating, leading to a decrease in the body’s effective circulating blood volume and hemoconcentration, which ultimately lead to the occurrence of cerebrovascular diseases. (2) Hyperthyroidism is an autoimmune disease characterized by the production of various autoantibodies. Moodley *et al.* confirmed that there are antigen targets of thyroid autoantibodies in cerebral blood vessels, causing an inflammatory response in the blood vessel wall. These result in damage to vascular endothelial cells, platelet adhesion, and aggregation to form thrombus, leading to cerebral infarction.^[20,21]^ In addition, Zhang *et al.* showed that elevated levels of thyroid antibodies are associated with intracranial artery stenosis.^[22]^ (3) Hyperthyroidism can shorten activated partial thromboplastin time, increase fibrinogen levels, increase the activity of factor VIII and factor X, cause blood hypercoagulability, and promote the formation of intravascular microemboli.^[8,9,23,24]^ (4) The incidence of atrial fibrillation in hyperthyroidism is 13.8%, whereas atrial fibrillation was observed in up to 40% of patients with TS.^[25]^ Chaker *et al.* found that a higher concentration of thyroxine (FT4) was associated with a higher incidence of atrial fibrillation. During a thyroid storm, the body secretes a large amount of thyroid hormones. Therefore, cerebral infarction caused by atrial fibrillation should be considered.^[26]^ This patient had a history of hyperthyroidism for 2 years, had not been treated regularly, and had a smoking history of 16 years. We speculate that long-term chronic inflammatory stimulation leads to vascular endothelial damage and sclerosis. Infection induces a thyroid storm, which may lead to hemoconcentration, vasospasm, and ultimately, acute cerebral infarction. However, the relationship between thyroid storm and stroke is not completely clear, and the present study has limitations. It cannot be ruled out that the co-occurrence of thyroid storm and acute cerebral infarction in this patient is incidental. Therefore, more research is still needed to clarify the pathogenesis of thyroid storm complicated with ischemic stroke in the future.

The blood supply to the corpus callosum is rich, mainly including the overlapping blood supply of the pericallosal, anterior cerebral, posterior cerebral, anterior communicating, and posterior choroidal arteries.^[27]^ The corpus callosum is rich in collateral circulation; therefore, corpus callosum infarction is rare in clinical practice. Li et al found that 59 of 1629 patients with ischemic stroke had corpus callosum infarction, with an incidence rate of 3.6%.^[28]^ A corpus callosum infarction usually indicates extensive arteriosclerosis and multivessel diseases.^[27]^ The head MRA of this patient showed partial visualization of the basilar artery and bilateral vertebral arteries, and slight visualization of the proximal end of the right anterior cerebral artery A2 segment. Vascular lesions affect both the anterior and posterior circulation, resulting in infarction of the corpus callosum. Acute corpus callosum infarction mostly manifests as limb movement disorders, language disorders, or cognitive and mental disorders. This patient had left hemiplegia with slurred speech, consistent with the clinical features of acute corpus callosum infarction.

Thyroid storm is a life-threatening disease, with a mortality rate > 10%.^[2]^ If a thyroid storm is complicated by an acute cerebral infarction, the mortality rate is higher. Therefore, as soon as a thyroid storm is suspected, treatment should be initiated immediately. Treatment for TS mainly comprises agents that suppress excessive thyroid hormones, including anti-thyroid drugs (ATDs) and iodine. In addition, beta-blockers and glucocorticoids are also used in the clinical treatment of TS.^[29]^ For patients who cannot tolerate drugs or have failed drug therapy, alternative treatments need to be considered, including the use of extracorporeal systems such as continuous renal replacement therapy (CRRT), veno-arterial extracorporeal membrane oxygenation (VA-ECMO), and therapeutic plasma exchange (TPE), etc.^[12]^ The recommended doses of glucocorticoids in this condition are hydrocortisone 100 mg every 6–8 h (300–400 mg/d) or dexamethasone 2 mg intravenous injection every 6 h (8 mg/d) continued until the resolution of the TS.^[30]^ However, a recent large-scale data analysis found that routine use of corticosteroids did not improve survival in patients with TS. This suggests that clinicians should consider the use of glucocorticoids on an individual patient basis, taking into account the risk of infection and hyperglycemia.^[29]^ Successful treatment of thyroid storm depends on early suppression of thyroid hormone secretion and suppression of peripheral T4 to T3 transition. Some scholars have observed that after the thyroid hormone levels in patients with thyroid storm complicated by acute cerebral infarction returned to normal following treatment, cerebral artery angiography showed that the stenosis site also returned to normal, and the neurological damage of the patients was reversible with the improvement of hyperthyroidism.^[31]^ This indicates that the treatment of thyroid storm is key to improving the condition. In this case, the symptoms of cerebral infarction gradually improved with improvement of the thyroid storm. In addition to TS complicated with acute cerebral infarction, there are also a small number of reports on thyroid storm complicated with other emergencies. Some scholars have reported cases of diabetic ketoacidosis complicated with TS.^[1,32]^ And some scholars have reported cases of head and neck trauma or surgery-induced TS.^[6]^

It is worth noting that inflammation may play an important role in the occurrence and development of thyroid storm and acute cerebral infarction. During thyroid storm, the body may acutely secrete and release high levels of pro-inflammatory cytokines, resulting in a severe inflammatory response. Studies have shown that pyruvate kinase M2 (PKM2) plays an important role in the development of some inflammatory diseases, and PKM2-mediated immunometabolic reprogramming promotes the release of a large number of pro-inflammatory cytokines, causing excessive inflammatory responses. Therefore, PKM2 may be a potential therapeutic target for the treatment of related inflammatory diseases and may have therapeutic implications for the inflammatory response that occurs during thyroid storm.^[33]^ Resident immune cells (microglia) in the brain are activated during acute cerebral infarction and release a variety of proinflammatory cytokines and reactive oxygen species (ROS), which may further aggravate thyroid crisis. Normalizing the aberrant inflammatory activation of microglia may be a potential therapeutic target for obtaining a better outcome after cerebral infarction.^[34]^ In addition, some scholars have found that patients with other thyroid diseases such as Hashimoto’s thyroiditis and thyroid nodules have increased inflammatory markers. The neutrophil-to-lymphocyte ratio (NLR) is a new marker of inflammation, and Gulali et al suggested that increased NLR or red blood cell distribution width (RDW) may serve as an indicator of Hashimoto’s thyroiditis.^[35,36]^ Elevated mean platelet volume (MPV) can be used as an auxiliary diagnostic tool to differentiate benign and malignant thyroid nodules.^[37]^

In summary, careful physical examination should be performed in patients with thyroid storm, and head imaging examination should be improved for the early detection of cerebral infarction. Simultaneously, patients with a history of hyperthyroidism should be regularly screened for cerebrovascular disease and timely intervention to reduce the possibility of cerebrovascular disease or its severity should be considered. For young patients with stroke, routine screening of thyroid function should be performed, and active treatment should be administered as soon as possible.

## Acknowledgments

The authors are very grateful to the patient for his kind contribution to this study.

## Author contributions

Conceptualization: Yunjia Cai, Linan Ren, Xiaokun Gang, Guixia Wang.

Data curation: Yunjia Cai.

Formal analysis: Yunjia Cai.

Funding acquisition: Guixia Wang.

Investigation: Xinming Liu, Chen Li.

Methodology: Linan Ren, Xiaokun Gang.

Project administration: Guixia Wang.

Resources: Xiaokun Gang, Guixia Wang.

Validation: Xiaokun Gang, Guixia Wang.

Visualization: Guixia Wang.

Writing – original draft: Yunjia Cai.

Writing – review & editing: Linan Ren.
